# Lean Women on Metformin and Oral Contraceptives for Polycystic Ovary Syndrome Demonstrate a Dehydrated Osteosarcopenic Phenotype: A Pilot Study

**DOI:** 10.3390/nu11092055

**Published:** 2019-09-02

**Authors:** Charikleia Stefanaki, Flora Bacopoulou, Eleni Kandaraki, Dario Boschiero, Evanthia Diamandi-Kandarakis

**Affiliations:** 1 Center for Adolescent Medicine and UNESCO Chair on Adolescent Health Care, First Department of Pediatrics, School of Medicine, National and Kapodistrian University of Athens, Aghia Sophia Children’s Hospital, 11527 Athens, Greece; 2 Department of Endocrinology, Diabetes and Metabolism, Hygeia Hospital, 15123 Athens, Greece; 3 BIOTEKNA Biomedical Technologies, 30020 Venice, Italy

**Keywords:** bio-impedance, polycystic ovary syndrome, pcos, osteosarcopenia, body composition, metformin, oral contraceptives

## Abstract

Scarce data exist on the body composition of lean women with polycystic ovary syndrome (PCOS) on treatment with metformin and oral contraceptives (OCs). Thirty-four lean (body mass index 18.5–24.9 kg/m^2^) women (17 with PCOS on metformin and OCs treatment for six months and 17 controls) aged 18–40 years were assessed for body composition parameters (fat, muscle, glycogen, protein masses, bone masses, and body water compartments) and phase angles. PCOS patients demonstrated lower muscle, glycogen and protein masses (U = 60, *p* = 0.003), along with a lower bone mineral content and mass (U = 78, *p* = 0.021; U = 74, *p* = 0.014) than their healthy counterparts, while total and abdominal fat masses were similar between the two groups. PCOS patients also exhibited increased extracellular body water (U = 10, *p* < 0.001) and decreased intracellular water, compatible with low-grade inflammation and cellular dehydration. Key differences in body composition between women with PCOS and controls demonstrated an osteosarcopenic body composition phenotype in PCOS patients. A confirmation of these findings in larger studies may render osteosarcopenia management a targeted adjunct therapy in women with PCOS.

## 1. Introduction

Polycystic ovary syndrome (PCOS) is a multifarious endocrinopathy affecting up to 15% of reproductive-aged women worldwide [1] and 5%–7% of Greek women, with adverse consequences for their health and quality of life [2]. The syndrome has been studied extensively in terms of genetics, epigenetics, hormonal interactions, diagnosis and complications. However, more robust studies are needed to explore the mechanisms accountable for the improvements after dietary or pharmacological interventions [3]. PCOS phenotypes are stratified according to age, reproductive status and body mass index [4]. Body mass index (BMI) is an inadequate means of body composition assessment, as it measures the degree of excess body weight but not the lean and fat body masses, both of which influence morbidity and mortality [5]. Moreover, there is debate about the lean phenotype of PCOS regarding the characteristics and management modalities [6,7].

A bioelectrical impedance (bio-impedance) analysis (BIA) provides an assessment of body composition based on the varying bioelectric resistance of different tissues [8]. It is safe, inexpensive, patient-friendly and convenient. A BIA assesses total body water volume, fat-free mass [9], skeletal muscle mass [10], mineral distribution between different body compartments and cell membrane functional integrity [11].

Lately, evidence has accumulated about the effect of stress and, consequently, about subclinical, low-grade inflammation on body composition [12,13]. Additionally, a significant number of studies have attributed significant anti-inflammatory [14] and antioxidant properties to metformin, along with a reduction in body fat in individuals with hyperglycemic states [15,16,17,18].

Increased salivary cortisol and α-amylase concentrations have been reported in PCOS patients which, compared with healthy counterparts, demonstrated a sustained low-grade inflammation status [12,19,20]. PCOS is associated with an increased glucocorticoid receptor protein concentration and hypothalamic–pituitary–adrenal (HPA) axis upregulation and oversensitivity [21]. These findings suggest an association between the stress markers, metabolism and alterations in body composition parameters of PCOS patients [22]. The combination of oral metformin and oral contraceptives is one of the most common treatments for PCOS [5,23]. The administration of the insulin-sensitizer metformin has resulted in significant reductions of visceral and subcutaneous adipose tissues [24,25], whereas oral contraceptives help in the management of both menstrual disorders and androgen excess symptoms [23]. Therefore, it is expected that the combination of metformin and oral contraceptives (OCs) may help in maintaining the homeostasis of body composition in these women [26].

There are few bio-impedance studies that have assessed body composition in lean women with PCOS [27,28], exhibiting inconclusive or conflicting results [29,30,31]. Data are even more scarce for lean PCOS patients of reproductive age on treatment with metformin and OCs. The aim of this pilot study was to assess potential differences in body composition parameters between reproductive age lean patients with PCOS on combined treatment with OCs and metformin, as well as healthy age- and BMI-matched controls.

## 2. Materials and Methods

### 2.1. Study Design—Setting—Participants

The study was approved by the Athens University Medical School Ethics Committee and was in accordance with the Helsinki Declaration of the World Medical Association for human studies (Repository number: 345/28-8-2012). All participants gave informed consent after an explanation of the purpose and nature of every study procedure. The study took place at the Unit of Translational and Clinical Research in Endocrinology of the National and Kapodistrian University of Athens (‘study site’) in one academic year period (from September 2013 to June 2014).

Study participants included a convenience sample of lean (BMI 18.5–24.9 kg/m^2^) females of reproductive age (18–40 years) with polycystic ovary syndrome (PCOS group) and clinically healthy age- and BMI-matched females (control group). The PCOS group included women with an established diagnosis of PCOS according to the Rotterdam criteria (the presence of at least two of the three diagnostic criteria, i.e., chronic anovulation, clinical and/or biochemical hyperandrogenism, and polycystic ovaries on ultrasound—after the exclusion of related disorders) [5]. PCOS patients received OCs and metformin for 6 months. The control group included healthy, euthyroid women with a menstrual cycle of 26–35 days who were on no medication. Participants visited the study site mostly for a routine check-up.

Exclusion criteria included pregnancy, chronic life-threatening diseases, epilepsy, other neurological or severe mental disorders, and the presence of metal devices such as orthopedic prostheses or pacemakers that may intervene with BIA measurement evaluations. Women with a history of atopic dermatitis were not included in the study to avoid irritation from the skin patches used in BIA-ACC measurements.

### 2.2. Variable Measurements

#### BIA Measurements

The body composition bio-impedance analysis was performed with a BIA-ACC device (BIOTEKNA Biomedical Technologies) [32,33]. This device applies alternating currents using two different frequencies, 50 and 1.5 kHz [bi-frequency measurement method], to measure body composition based on a multi-compartment model (2C, 3C, 4C, 5C). Study participants lied supine on an electrically non-conductive flat surface, and there was no contact with metallic elements. Two electrodes were applied on the dorsal surface of the right hand, and two electrodes were applied on the dorsal surface of the right foot of each participant. The formulas used for computations have been previously described in detail. This method is simple, rapid, does not require skilled staff and does not expose individuals to radiation [32,33]. The device has been validated against dual energy X-ray absorptiometry (DXA) [33]. The measurements of the BIA included: Total body water in liters [TBW (L)], extracellular water as a percentage of TBW [ECW (%TBW)], intracellular water as a percentage of TBW [ICW (%TBW)], intracellular water in liters [ICW (L)], fat-free mass in kilograms [FFM (Kg)], fat mass as a percentage of weight [FM (%Weight)], fat mass in kilograms [FM (Kg)], phase angle in degrees °, total body proteins in kilograms [TBPro (Kg)], total body proteins as a percentage of fat-free mass [TBPro (%FFM)], glycogen in kilograms [Gly (Kg)], glycogen as a percentage of fat-free mass [Gly (%FFM)], body minerals in kilograms [Bm (Kg)], skeletal muscles in kilograms [Skeletal Muscles (Kg)], skeletal muscles as a percentage of fat-free mass [(%FFM)], bone mass in kilograms [Bones (Kg)], appendicular lean soft mass tissue in kilograms [ALST (Kg)], appendicular lean soft mass tissue as a percentage of fat-free mass [ALST (%FFM)], abdominal adipose tissue in cubic meters [AAT (cm^2^)], adipose tissue in kilograms [AT (Kg)], adipose tissue as a percentage of weight [AT (%Weight)], and the presence of sarcopenia [Sarcopenia].

### 2.3. Bias

The possibility of selection bias was addressed by the similarity in the recruitment of both groups.

### 2.4. Statistical Analyses and Sample Size Calculation

A two-sided *p*-value < 0.05 was considered significant in all analyses. Data normality was examined using the Shapiro–Wilk test. Descriptive statistics are presented by median values and by 25th and 75th percentiles. Due to the asymmetry of distributions and the small number of participants, Mann–Whitney U-tests were used to test the statistical significance of differences between the two groups. Kendall’s tau tests were used to evaluate correlations.

An effect size r calculation was also performed by dividing the z value by the squared root of the number of the total sample. The Holm’s method was used for adjusting the alpha level [34], using the ‘Holm–Bonferroni Sequential Correction: An EXCEL Calculator—Ver. 1.2′ form by Justin Gaetano [35]. As this study was a pilot, the aim was to include over 12 participants per group [36].

## 3. Results

### 3.1. Participants

A total of 102 women were initially screened, of which 52 were eligible to be included in the study. From these, 17 patients with PCOS and an equal number of clinically healthy controls, matched for age and BMI, agreed to participate.

### 3.2. Descriptive Data

The baseline characteristics of the participants of the study did not differ between the two groups, as shown in Table 1.

### 3.3. Outcome Data—Main Results

**Figure 1:**
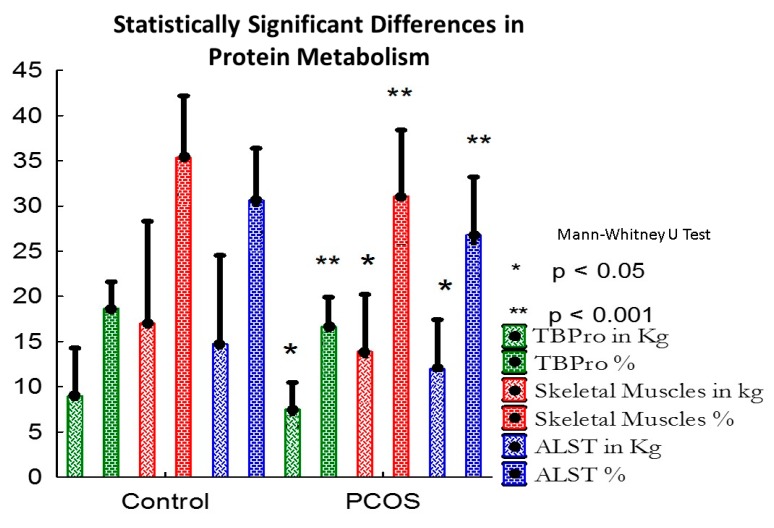
Statistically significant differences in protein metabolism.

The PCOS group demonstrated sarcopenia (x^2^ = 17.486, *df* = 33, *p* < 0.001) with a low skeletal muscle mass measured both in kilograms (*p* = 0.004, r = 0.5) and as a percentage of fat free mass (*p* < 0.001, r = 0.7). On the other hand, the fat free mass (FFM), in kilograms (kg) was also lower in patients with PCOS than controls, but the size effect was moderate (*p* = 0.05, r = 0.33). The glycogen mass as a percentage of FFM and in kilograms was significantly decreased in the PCOS group compared to those of the control group (*p* < 0.001, r = 0.73; *p* = 0.004, r = 0.5, respectively), along with decreased total body protein measured in kg and as a percentage of the fat free mass when compared with the control group (*p* = 0.003, r = 0.5; *p* < 0.001, r = 0.74, respectively). Appendicular lean soft tissue in kg and as %FFM was also decreased in the PCOS patients vs. controls (*p* = 0.003, r = 0.5; *p* < 0.001, r = 0.69, respectively), as shown in Figure 1. The total adipose tissue mass (*ns*, r = 0.063) and the abdominal fat mass (*ns*, r = 0.06) did not differ significantly between the two groups.

**Figure 2:**
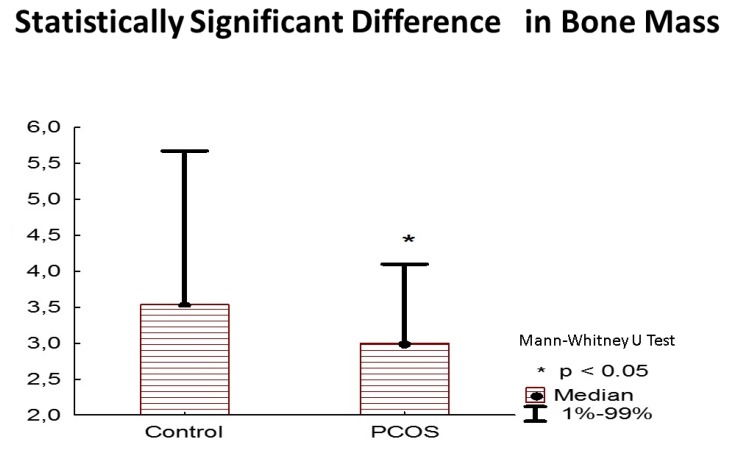
Statistically significant difference in bone mass in kilograms.

The bone mineral content and bone mass were decreased in the PCOS group compared with the control group (*p* = 0.021, r = 0.4; *p* = 0.014, r = 0.41, respectively), as shown in Figure 2. The PCOS group was also “dehydrated” in terms of total body water volume and intracellular body water expressed as a percentage of total body water and in liters (*p* = 0.026, r = 0.378; *p* < 0.001, r = 0.799; *p* = 0.0025, r = 0.52, respectively) compared with the control group.

Extracellular body water, expressed as a percentage of total body water, was increased in the PCOS group (*p* < 0.001, r = −0.8). A statistically significant difference in the phase angle was also observed between the two groups, with the PCOS group demonstrating lower degrees (*p* < 0.001, r = 0.71).

### 3.4. Other Analyses

No statistically significant correlations were observed between the body composition variables assessed by the BIA-ACC device and the participants’ characteristics, either in the total sample or in the two groups separately.

## 4. Discussion

Our study revealed statistically significant differences in the body composition between lean women of reproductive age with PCOS under treatment with metformin and oral contraceptives and that of control women. Patients with PCOS demonstrated a decreased fat-free mass (decreased skeletal muscle mass—sarcopenia) [37], a decreased bone mineral content and bone mass, decreased total body water and intracellular body water. Conclusively, lean PCOS patients of reproductive age receiving metformin and OCs for six months presented with osteosarcopenic elements and cellular dehydration.

Dolfing et al., with the use of a BIA, compared lean PCOS patients with matched controls and found similar lean body masses between the two groups. However, the results of this study were based on a very small sample [28]. PCOS is a hyperandrogenic disorder, and androgens are known to increase lean and muscle mass in males. A significant positive correlation between serum androgen levels and muscle mass has also been reported in patients with PCOS [38]. Nevertheless, another study has demonstrated that in women with PCOS, increases in lean mass are associated with insulin resistance and central obesity rather than with energy intake, physical activity or androgens [39]. The PCOS group in our study was under treatment with metformin. It has been shown that metformin improves not only insulin resistance but also biochemical hyperandrogenemia [40]. No data exist regarding the effect of oral contraceptives on the body composition of lean PCOS patients, but in menopausal females, OCs seem to induce muscle anabolism [41].

Vgontzas et al. reported a significant increase of circulating interleukin-6 [42], while Ciaraldi et al. demonstrated a low-grade, systemic inflammation [43] in both lean and obese PCOS patients. Low-grade inflammation is a major cause of skeletal muscle wasting [44], even in the presence of elevated androgen concentrations [45]. A study by Ibfelt et al. showed that inflammatory cytokines directly induce insulin resistance in skeletal muscles [46]. Additionally, a recent study correlated low-grade inflammation with osteosarcopenic elements in a large sample of healthy overweight/obese individuals [47].

Thus, it seems that PCOS, apart from other characteristics, is a disorder of low-grade inflammation, as reflected in the body composition of women in this study and in accordance with recent studies [12,13]. It is known that modifications of the mitochondrial system influence the production of reactive oxygen species that play important role in muscle function. Additionally, dysfunctional mitochondria trigger catabolic signaling pathways which feed-forward to the nucleus to promote the activation of muscle atrophy. Our results seem to reinforce these findings [48].

Recent data suggest the contribution of hepatic disorders, such as the non-alcoholic fatty liver disease (NAFLD), to PCOS phenotypes [49,50]. The PCOS group in this study was under treatment with metformin and OCs. Data from previous reports indicate that the diminished hepatic glucose output observed with metformin may result from the inhibition of electron transport in mitochondrial respiratory Complex I [51], as well as from the antagonism of glucagon action in the liver [52]. Another study also demonstrated the loss of fat free mass due to the weight loss attributed to metformin therapy [53].

In several studies [37,54,55,56], metformin administration in type 2 diabetic patients led to an increase of serum fibroblast growth factor 21 (FGF21) concentrations; a hormone increased in muscle wasting and degeneration. Additionally, Cetrone et al. concluded that AMP-activated protein kinase (AMPK) agonists, like metformin, might induce muscle mass atrophy compatible with the reduced muscle mass of the PCOS women [57]. However, in these studies, PCOS patients were overweight or obese. Thus, a direct comparison with the results of our study is not feasible. Finally, the increased muscle sympathetic nerve activity in PCOS patients [58] may induce muscle degradation [59]. Another potential contributor to the decreased muscle mass in PCOS patients might be the general lack of physical exercise, possibly associated with the depressive symptomatology of these patients [60] or with their obstructive sleep apnea, as shown in some studies [61].

No statistically significant differences were found in the fat mass between the PCOS and the control groups of this study. Similarly, Durmus et al. found a higher visceral adiposity in overweight and/or obese PCOS patients compared to peer controls and non-obese PCOS patients [62]. Lean women with PCOS had a similar total fat mass to that of healthy BMI-matched controls in two studies [27,28], also confirming our results. The fat mass and abdominal adipose tissue (measured in cm^2^) did not differ between the two groups in this study. Visceral adiposity is a marker of additional metabolic risk in polycystic ovary syndrome; however, this feature may be more evident in overweight and/or obese PCOS patients [63], which were not included in our study. Moreover, the PCOS group of our study was on treatment, which might have induced a loss of fat mass [25,43,64] and an amelioration of their endothelial function [65].

Hyperandrogenism and hyperinsulinemia, well-recognized characteristics of PCOS, may influence bone mineral density and biochemical markers of bone turnover. Bone formation markers were found to be decreased in younger PCOS patients compared with healthy controls [66]. It has also been reported that metformin and sulfonylureas may have a neutral or positive effect on bone health [67,68]. Furthermore, low osteocalcin concentrations in PCOS have been reported, possibly suggesting the under-functioning of osteoblasts in these patients [69]. The bone mass and bone mineral content were decreased in the PCOS group, and bone mineral density has been found to be lower in lean patients than in matched controls [70]. In the cohort study of Lingaiah et al., metformin treatment, when compared with a placebo, was related with reduced bone turnover, as suggested by reductions in markers of bone formation and resorption, leading to slower bone remodeling in premenopausal women with PCOS [71].

Women with PCOS in this study exhibited shifts in body water compartments. They were overall ’dehydrated,’ but also had an augmented extracellular water compartment. Again, several studies have reported low-grade inflammation in PCOS [72,73,74], even during metformin therapy [75], possibly increasing osmolality as a result of the increased concentrations of inflammatory mediators [76]. When systemic, low-grade inflammation is present, the microcirculation and membrane permeability of the blood vessels are disrupted, resulting in body water compartment shifts, even in the presence of overall dehydration [77]. It should be noted that OCs do not provoke water retention, as was once, believed [78], which implies no contribution of these agents to the observed increased extracellular water in our patients [79,80]. Furthermore, recent studies have established that OCs do not induce changes in body water compartments [64,81].

A statistically significant difference in the phase angle was also observed between the two groups of our study. The phase angle represents the angle between the electric reactance and resistance, as it depends on cell membrane integrity and body cell mass. A low-phase angle is consistent with low reactance, associated with either cell death or breakdown in the selective permeability of the cell membrane. Generally, a phase angle is lower in disease than in health, increasing with an improvement of the clinical status. A high level of inflammatory and oxidative stress in our patients associated with their low-grade inflammation might explain the findings related to the phase angle [82].

The limitations of the study include the small sample size [83], the cross-sectional nature, the lack of inclusion of other PCOS populations (e.g., PCOS patients not on treatment or obese PCOS patients) or non-PCOS populations on metformin and OCs, as well as the lack of inflammatory and muscle wasting biochemical markers [84] to support the body composition findings.

We demonstrated a decreased muscle mass, major shifts of the water compartments, and an overall “osteosarcopenic” phenotype of our lean PCOS patients under a six month treatment with metformin and OCs. All the elements of the disturbed body composition in osteosarcopenic PCOS patients may be future targets of therapeutic interventions, like the GLP-1 agonists [85]. Further longitudinal cohort studies with a larger number of patients are necessary to confirm our results.

## Figures and Tables

**Table 1:** Clinical profile of the sample and their bioelectrical impedance analysis (BIA) measurements.

Characteristics	Patients (n = 17)	Controls (n = 17)	Statistical Tests, *p* Value	Holm’s-Adjusted Alpha Levels	Rank	Outcome of Holm’s Adjusted Alpha Levels
	$\tilde{x}$ (25th–75th Percentiles)	$\tilde{x}$ (25th–75th Percentiles)				
**Age (years)**	27 (22–29)	26 (22–28)	M-W U test = 134, *p* = 0.73	0.199	12	Non-significant
**Clinical and Biochemical Hyperandrogenism**	15 (88.23%)	-		-		
**Polycystic ovaries on Ultrasound**	12 (70.58%)	-		-		
**Menstrual Problems**	14 (82.35%)	-		-		
**Metformin + OCPs**	17 (100%)	-		-		
**BMI**	22 (19.7–24.5)	22 (20.43–24.51)	M–W U test = 136, *p* = 0.76	0.199	13	Non-significant
**TBW (L)**	27.8 (27.3–29.3)	31.2(28.2–34.9)	M–W U test = 80.5, *p* = 0.026	0.110	4	Significant
**ECW (%TBW)**	47 (46–49)	42.5 (40.16–43.22)	M–W U test = 10, *p* < 0.001	0.082	1	Significant
**ICW (%TBW)**	53 (51–54)	57.5 (56.78–59.84)	M–W U test = 10, *p* < 0.001	0.082	1	Significant
**ICW (L)**	14.9 (13.9–15.8)	18 (15.8–21.1)	M–W U test = 57, *p* = 0.0025	0.099	3	Significant
**FFM (Kg)**	44.5 (43.1–46.8)	47.3 (44.3–52.6)	M–W U test = 87, *p* = 0.05	0.165	8	Significant
**FM (%Weight)**	27 (19–31)	26.27 (21.83–27.47)	M–W U test = 133, *p* = 0.69	0.199	9	Non-significant
**FM (Kg)**	13 (9.7–20.9)	17.9 (12.5–19.2)	M–W U test = 133, *p* = 0.70	0.199	10	Non-significant
**Phase Angle (degrees °)**	3.4 (2.6–4.2)	6.9 (5.8–8.4)	M–W U test = 24, *p* < 0.001	0.082	1	Significant
**TBPro (Kg)**	7.51 (7.05–7.95)	8.991 (7.94–10.47)	M–W U test = 59, *p* = 0.003	0.124	5	Significant
**TBPro (%FFM)**	16.67 (16.4–17.47)	18.692 (17.8–19.91)	M–W U test = 19, *p* < 0.001	0.082	1	Significant
**Gly (Kg)**	0.331 (0.31–0.35)	0.4 (0.35–0.46)	M–W U test = 60, *p* = 0.004	0.142	6	Significant
**Gly (%FFM)**	0.73 (0.72–0.77)	0.82 (0.78–0.88)	M–W U test = 20, *p* < 0.001	0.082	1	Significant
**Bm (Kg)**	1.64 (1.61–1.74)	1.91 (1.69–2.09)	M–W U test = 78, *p* = 0.021	0.090	2	Significant
**Skeletal Muscles (Kg)**	13.9 (12.9–14.8)	17.04 (14.79–20.2)	M–W U test = 60, *p* = 0.004	0.142	6	Significant
**Skeletal Muscles (%FFM)**	31.1 (29.3–32.4)	35.37 (33.16–38.4)	M–W U test = 28, *p* < 0.001	0.082	1	Significant
**Bones (Kg)**	3 (3–3.2)	3.53 (3.13–3.88)	M–W U test = 74, *p* = 0.014	0.165	7	Significant
**ALST (Kg)**	12 (11.1–12.7)	14.74 (12.72–17.44)	M–W U test = 58, *p* = 0.003	0.110	5	Significant
**ALST (%FFM)**	26.8 (25.1–28.1)	30.64 (28.52–33.16)	M–W U test = 27, *p* < 0.001	0.082	1	Significant
**AAT (cm^2^)**	191.2 (135.6–324.5)	273.88 (182.82–295.8)	M–W U test = 134, *p* = 0.71	0.199	11	Non-significant
**AT (Kg)**	16.3 (12.1–26.1)	22.38 (15.63–24)	M–W U test = 133, *p* = 0.70	0.199	10	Non-significant
**AT (%Weight)**	33.2 (23.9–39)	32.84 (27.29–34.34)	M–W U test = 134, *p* = 0.71	0.199	11	Non-significant
**Sarcopenia**	13/17 (76.47%)	1/17 (5.88%)	x^2^ = 3414.167, *df* = 33, *p* < 0.001, RR = 4.643, 95% CI: 1.985–6.526	Significant		

Abbreviations: M–W U test = Mann–Whitney U test, x^2^ = Chi-squared test, *p* = *p*-value, $\tilde{x}$ = Median value, OCP = Oral contraceptive pill, BMI = Body mass index, TBW (%Weight) = Total body water (as percent of weight), ECW = Extracellular water, ICW = Intracellular water, FFM = Fat free mass, FM = Fat mass, TBPro = Total body protein, Gly = Glycogen, Bm = Bone minerals, ALST = Appendicular lean soft tissue, AAT = Abdominal adipose tissue, AT = Adipose tissue.

## References

[1] Fauser B.C., Tarlatzis B.C., Rebar R.W., Legro R.S., Balen A.H., Lobo R., Carmina E., Chang J., Yildiz B.O., Laven J.S. (2012). Consensus on women’s health aspects of polycystic ovary syndrome (PCOS): The Amsterdam ESHRE/ASRM-Sponsored 3rd PCOS Consensus Workshop Group. Fertil. Steril..

[2] Diamanti-Kandarakis E., Kouli C.R., Bergiele A.T., Filandra F.A., Tsianateli T.C., Spina G.G., Zapanti E.D., Bartzis M.I. (1999). A survey of the polycystic ovary syndrome in the Greek island of Lesbos: Hormonal and metabolic profile. J. Clin. Endocrinol. Metab..

[3] Jeanes Y.M., Reeves S. (2017). Metabolic consequences of obesity and insulin resistance in polycystic ovary syndrome: Diagnostic and methodological challenges. Nutr. Res. Rev..

[4] Komarowska H., Stangierski A., Warmuz-Stangierska I., Lodyga M., Ochmanska K., Wasko R., Wanic-Kossowska M., Ruchala M. (2013). Differences in the psychological and hormonal presentation of lean and obese patients with polycystic ovary syndrome. Neuro Endocrinol. Lett..

[5] Legro R.S., Arslanian S.A., Ehrmann D.A., Hoeger K.M., Murad M.H., Pasquali R., Welt C.K., Endocrine S. (2013). Diagnosis and treatment of polycystic ovary syndrome: An Endocrine Society clinical practice guideline. J. Clin. Endocrinol. Metab..

[6] Toosy S., Sodi R., Pappachan J.M. (2018). Lean polycystic ovary syndrome (PCOS): An evidence-based practical approach. J. Diabetes Metab. Disord..

[7] Goyal M., Dawood A.S. (2017). Debates Regarding Lean Patients with Polycystic Ovary Syndrome: A Narrative Review. J. Hum. Reprod. Sci..

[8] Health, N.I.O. Bioelectrical Impedance Analysis in Body Composition Measurement. http://consensus.nih.gov/1994/1994BioelectricImpedanceBodyta015html.htm.

[9] Gupta D., Lammersfeld C.A., Burrows J.L., Dahlk S.L., Vashi P.G., Grutsch J.F., Hoffman S., Lis C.G. (2004). Bioelectrical impedance phase angle in clinical practice: Implications for prognosis in advanced colorectal cancer. Am. J. Clin. Nutr..

[10] Rubbieri G., Mossello E., Di Bari M. (2014). Techniques for the diagnosis of sarcopenia. Clin. Cases Miner. Bone Metab. Off. J. Ital. Soc. Osteoporos. Miner. Metab. Skelet. Dis..

[11] Jebb S.A., Siervo M., Murgatroyd P.R., Evans S., Fruhbeck G., Prentice A.M. (2007). Validity of the leg-to-leg bioimpedance to estimate changes in body fat during weight loss and regain in overweight women: A comparison with multi-compartment models. Int. J. Obes..

[12] Basu B.R., Chowdhury O., Saha S.K. (2018). Possible Link Between Stress-related Factors and Altered Body Composition in Women with Polycystic Ovarian Syndrome. J. Hum. Reprod. Sci..

[13] Stefanaki C., Pervanidou P., Boschiero D., Chrousos G.P. (2018). Chronic stress and body composition disorders: Implications for health and disease. Hormones.

[14] Cameron A.R., Morrison V.L., Levin D., Mohan M., Forteath C., Beall C., McNeilly A.D., Balfour D.J., Savinko T., Wong A.K. (2016). Anti-Inflammatory Effects of Metformin Irrespective of Diabetes Status. Circ. Res..

[15] Du K., Ramachandran A., Weemhoff J.L., Chavan H., Xie Y., Krishnamurthy P., Jaeschke H. (2016). Editor’s Highlight: Metformin Protects Against Acetaminophen Hepatotoxicity by Attenuation of Mitochondrial Oxidant Stress and Dysfunction. Toxicol. Sci. Off. J. Soc. Toxicol..

[16] Kurutas E.B. (2016). The importance of antioxidants which play the role in cellular response against oxidative/nitrosative stress: Current state. Nutr. J..

[17] Balamash K.S., Alkreathy H.M., Al Gahdali E.H., Khoja S.O., Ahmad A. (2018). Comparative Biochemical and Histopathological Studies on the Efficacy of Metformin and Virgin Olive Oil against Streptozotocin-Induced Diabetes in Sprague-Dawley Rats. J. Diabetes Res..

[18] Zhao H., Lai Q., Zhang J., Huang C., Jia L. (2018). Antioxidant and Hypoglycemic Effects of Acidic-Extractable Polysaccharides from Cordyceps militaris on Type 2 Diabetes Mice. Oxidative Med. Cell. Longev..

[19] Stefanaki C., Bacopoulou F., Livadas S., Kandaraki A., Karachalios A., Chrousos G.P., Diamanti-Kandarakis E. (2015). Impact of a mindfulness stress management program on stress, anxiety, depression and quality of life in women with polycystic ovary syndrome: A randomized controlled trial. Stress.

[20] Chatzigeorgiou A., Kandaraki E., Piperi C., Livadas S., Papavassiliou A.G., Koutsilieris M., Papalois A., Diamanti-Kandarakis E. (2013). Dietary glycotoxins affect scavenger receptor expression and the hormonal profile of female rats. J. Endocrinol..

[21] Milutinovic D.V., Macut D., Bozic I., Nestorov J., Damjanovic S., Matic G. (2011). Hypothalamic-pituitary-adrenocortical axis hypersensitivity and glucocorticoid receptor expression and function in women with polycystic ovary syndrome. Exp. Clin. Endocrinol. Diabetes Off. J. Ger. Soc. Endocrinol. Ger. Diabetes Assoc..

[22] Diamanti-Kandarakis E., Katsikis I., Piperi C., Kandaraki E., Piouka A., Papavassiliou A.G., Panidis D. (2008). Increased serum advanced glycation end-products is a distinct finding in lean women with polycystic ovary syndrome (PCOS). Clin. Endocrinol..

[23] Conway G., Dewailly D., Diamanti-Kandarakis E., Escobar-Morreale H.F., Franks S., Gambineri A., Kelestimur F., Macut D., Micic D., Pasquali R. (2014). The polycystic ovary syndrome: A position statement from the European Society of Endocrinology. Eur. J. Endocrinol..

[24] Pasquali R., Gambineri A., Biscotti D., Vicennati V., Gagliardi L., Colitta D., Fiorini S., Cognigni G.E., Filicori M., Morselli-Labate A.M. (2000). Effect of long-term treatment with metformin added to hypocaloric diet on body composition, fat distribution, and androgen and insulin levels in abdominally obese women with and without the polycystic ovary syndrome. J. Clin. Endocrinol. Metab..

[25] Aghili R., Malek M., Valojerdi A.E., Banazadeh Z., Najafi L., Khamseh M.E. (2014). Body composition in adults with newly diagnosed type 2 diabetes: Effects of metformin. J. Diabetes Metab. Disord..

[26] Kujawska-Luczak M., Musialik K., Szulinska M., Swora-Cwynar E., Kargulewicz A., Grzymislawska M., Pupek-Musialik D., Bogdanski P. (2017). The effect of orlistat versus metformin on body composition and insulin resistance in obese premenopausal women: 3-month randomized prospective open-label study. Arch. Med. Sci..

[27] Aydin K., Cinar N., Aksoy D.Y., Bozdag G., Yildiz B.O. (2013). Body composition in lean women with polycystic ovary syndrome: Effect of ethinyl estradiol and drospirenone combination. Contraception.

[28] Dolfing J.G., Stassen C.M., van Haard P.M., Wolffenbuttel B.H., Schweitzer D.H. (2011). Comparison of MRI-assessed body fat content between lean women with polycystic ovary syndrome (PCOS) and matched controls: Less visceral fat with PCOS. Hum. Reprod..

[29] Hestiantoro A., Kapnosa Hasani R.D., Shadrina A., Situmorang H., Ilma N., Muharam R., Sumapraja K., Wiweko B. (2018). Body fat percentage is a better marker than body mass index for determining inflammation status in polycystic ovary syndrome. Int. J. Reprod. Biomed..

[30] Kogure G.S., Silva R.C., Miranda-Furtado C.L., Ribeiro V.B., Pedroso D.C.C., Melo A.S., Ferriani R.A., Reis R.M.D. (2018). Hyperandrogenism Enhances Muscle Strength After Progressive Resistance Training, Independent of Body Composition, in Women With Polycystic Ovary Syndrome. J. Strength Cond. Res..

[31] Un B., Dolapcioglu K.S., Guler Okyay A., Sahin H., Beyazit A. (2016). Evaluation of hs-CRP and visseral adiposity index in patients with policystic ovary syndrome by clinical and laboratory findings. Eur. J. Obstet. Gynecol. Reprod. Biol..

[32] Tsigos C., Stefanaki C., Lambrou G.I., Boschiero D., Chrousos G.P. (2015). Stress and inflammatory biomarkers and symptoms are associated with bioimpedance measures. Eur. J. Clin. Investig..

[33] Peppa M., Stefanaki C., Papaefstathiou A., Boschiero D., Dimitriadis G., Chrousos G.P. (2017). Bioimpedance analysis vs. DEXA as a screening tool for osteosarcopenia in lean, overweight and obese Caucasian postmenopausal females. Hormones.

[34] Holm S. (1979). A simple sequential rejective method procedure. Scand. J. Stat..

[35] Gaetano J. Dataset-Holm-Bonferroni Sequential Correction: An EXCEL Calculator—Ver. 1.2. https://www.researchgate.net/publication/242331583_Holm-Bonferroni_Sequential_Correction_An_EXCEL_Calculator_-_Ver._1.2.

[36] Julious S.A. (2005). Sample size of 12 per group rule of thumb for a pilot study. Pharmaceut. Stat..

[37] Kim K.H., Jeong Y.T., Kim S.H., Jung H.S., Park K.S., Lee H.Y., Lee M.S. (2013). Metformin-induced inhibition of the mitochondrial respiratory chain increases FGF21 expression via ATF4 activation. Biochem. Biophys. Res. Commun..

[38] Douchi T., Yamamoto S., Oki T., Maruta K., Kuwahata R., Nagata Y. (1999). Serum androgen levels and muscle mass in women with polycystic ovary syndrome. Obstet. Gynecol..

[39] Mario F.M., do Amarante F., Toscani M.K., Spritzer P.M. (2012). Lean muscle mass in classic or ovulatory PCOS: Association with central obesity and insulin resistance. Exp. Clin. Endocrinol. Diabetes Off. J. Ger. Soc. Endocrinol. Ger. Diabetes Assoc..

[40] Lundgren J.A., Kim S.H., Burt Solorzano C.M., McCartney C.R., Marshall J.C. (2018). Progesterone Suppression of Luteinizing Hormone Pulse Frequency in Adolescent Girls With Hyperandrogenism: Effects of Metformin. J. Clin. Endocrinol. Metab..

[41] Sorensen M.B., Rosenfalck A.M., Hojgaard L., Ottesen B. (2001). Obesity and sarcopenia after menopause are reversed by sex hormone replacement therapy. Obes. Res..

[42] Vgontzas A.N., Trakada G., Bixler E.O., Lin H.M., Pejovic S., Zoumakis E., Chrousos G.P., Legro R.S. (2006). Plasma interleukin 6 levels are elevated in polycystic ovary syndrome independently of obesity or sleep apnea. Metab. Clin. Exp..

[43] Ciaraldi T.P., Aroda V., Mudaliar S.R., Henry R.R. (2013). Inflammatory cytokines and chemokines, skeletal muscle and polycystic ovary syndrome: Effects of pioglitazone and metformin treatment. Metab. Clin. Exp..

[44] Degens H., Korhonen M.T. (2012). Factors contributing to the variability in muscle ageing. Maturitas.

[45] Bienso R.S., Olesen J., van Hauen L., Meinertz S., Halling J.F., Gliemann L., Plomgaard P., Pilegaard H. (2015). Exercise-induced AMPK and pyruvate dehydrogenase regulation is maintained during short-term low-grade inflammation. Pflug. Arch. Eur. J. Physiol..

[46] Ibfelt T., Fischer C.P., Plomgaard P., van Hall G., Pedersen B.K. (2014). The acute effects of low-dose TNF-alpha on glucose metabolism and beta-cell function in humans. Mediat. Inflamm..

[47] Stefanaki C., Peppa M., Boschiero D., Chrousos G.P. (2016). Healthy overweight/obese youth: Early osteosarcopenic obesity features. Eur. J. Clin. Investig..

[48] Romanello V., Sandri M. (2015). Mitochondrial Quality Control and Muscle Mass Maintenance. Front. Physiol..

[49] Carreau A.M., Pyle L., Garcia-Reyes Y., Rahat H., Vigers T., Jensen T., Scherzinger A., Nadeau K.J., Cree-Green M. (2019). Clinical prediction score of non-alcoholic fatty liver disease in adolescent girls with polycystic ovary syndrome (PCOS-HS index). Clin. Endocrinol..

[50] Lonardo A., Mantovani A., Lugari S., Targher G. (2019). NAFLD in Some Common Endocrine Diseases: Prevalence, Pathophysiology, and Principles of Diagnosis and Management. Int. J. Mol. Sci..

[51] El-Mir M.Y., Nogueira V., Fontaine E., Averet N., Rigoulet M., Leverve X. (2000). Dimethylbiguanide inhibits cell respiration via an indirect effect targeted on the respiratory chain complex I. J. Biol. Chem..

[52] Miller R.A., Chu Q., Xie J., Foretz M., Viollet B., Birnbaum M.J. (2013). Biguanides suppress hepatic glucagon signalling by decreasing production of cyclic AMP. Nature.

[53] Le Donne M., Alibrandi A., Giarrusso R., Lo Monaco I., Muraca U. (2012). Diet, metformin and inositol in overweight and obese women with polycystic ovary syndrome: Effects on body composition. Minerva Ginecol..

[54] Nygaard E.B., Vienberg S.G., Orskov C., Hansen H.S., Andersen B. (2012). Metformin stimulates FGF21 expression in primary hepatocytes. Exp. Diabetes Res..

[55] Kim K.H., Lee M.S. (2015). FGF21 as a mediator of adaptive responses to stress and metabolic benefits of anti-diabetic drugs. J. Endocrinol..

[56] Wessels B., Ciapaite J., van den Broek N.M., Nicolay K., Prompers J.J. (2014). Metformin impairs mitochondrial function in skeletal muscle of both lean and diabetic rats in a dose-dependent manner. PLoS ONE.

[57] Cetrone M., Mele A., Tricarico D. (2014). Effects of the antidiabetic drugs on the age-related atrophy and sarcopenia associated with diabetes type II. Curr. Diabetes Rev..

[58] Stener-Victorin E., Jedel E., Janson P.O., Sverrisdottir Y.B. (2009). Low-frequency electroacupuncture and physical exercise decrease high muscle sympathetic nerve activity in polycystic ovary syndrome. Am. J. Physiol. Regul. Integr. Comp. Physiol..

[59] Sandri M. (2008). Signaling in muscle atrophy and hypertrophy. Physiology.

[60] Banting L.K., Gibson-Helm M., Polman R., Teede H.J., Stepto N.K. (2014). Physical activity and mental health in women with polycystic ovary syndrome. BMC Women Health.

[61] Vgontzas A.N., Legro R.S., Bixler E.O., Grayev A., Kales A., Chrousos G.P. (2001). Polycystic ovary syndrome is associated with obstructive sleep apnea and daytime sleepiness: Role of insulin resistance. J. Clin. Endocrinol. Metab..

[62] Durmus U., Duran C., Ecirli S. (2017). Visceral adiposity index levels in overweight and/or obese, and non-obese patients with polycystic ovary syndrome and its relationship with metabolic and inflammatory parameters. J. Endocrinol. Investig..

[63] Ahmadi A., Akbarzadeh M., Mohammadi F., Akbari M., Jafari B., Tolide-Ie H.R. (2013). Anthropometric characteristics and dietary pattern of women with polycystic ovary syndrome. Indian J. Endocrinol. Metab..

[64] Glintborg D., Altinok M.L., Mumm H., Hermann A.P., Ravn P., Andersen M. (2014). Body composition is improved during 12 months’ treatment with metformin alone or combined with oral contraceptives compared with treatment with oral contraceptives in polycystic ovary syndrome. J. Clin. Endocrinol. Metab..

[65] Naka K.K., Kalantaridou S.N., Kravariti M., Bechlioulis A., Kazakos N., Calis K.A., Makrigiannakis A., Katsouras C.S., Chrousos G.P., Tsatsoulis A. (2011). Effect of the insulin sensitizers metformin and pioglitazone on endothelial function in young women with polycystic ovary syndrome: A prospective randomized study. Fertil. Steril..

[66] Lingaiah S., Morin-Papunen L., Piltonen T., Puurunen J., Sundstrom-Poromaa I., Stener-Victorin E., Bloigu R., Risteli J., Tapanainen J.S. (2017). Bone markers in polycystic ovary syndrome: A multicentre study. Clin. Endocrinol..

[67] Chandran M. (2017). Diabetes Drug Effects on the Skeleton. Calcif. Tissue Int..

[68] Adil M., Khan R.A., Kalam A., Venkata S.K., Kandhare A.D., Ghosh P., Sharma M. (2017). Effect of anti-diabetic drugs on bone metabolism: Evidence from preclinical and clinical studies. Pharmacol. Rep..

[69] Diamanti-Kandarakis E., Livadas S., Katsikis I., Piperi C., Mantziou A., Papavassiliou A.G., Panidis D. (2011). Serum concentrations of carboxylated osteocalcin are increased and associated with several components of the polycystic ovarian syndrome. J. Bone Miner. Metab..

[70] To W.W., Wong M.W. (2012). A comparison of bone mineral density in normal weight and obese adolescents with polycystic ovary syndrome. J. Pediatr. Adolesc. Gynecol..

[71] Lingaiah S., Morin-Papunen L., Risteli J., Tapanainen J.S. (2019). Metformin decreases bone turnover markers in polycystic ovary syndrome: A post hoc study. Fertil. Steril..

[72] Hefler-Frischmuth K., Walch K., Huebl W., Baumuehlner K., Tempfer C., Hefler L. (2010). Serologic markers of autoimmunity in women with polycystic ovary syndrome. Fertil. Steril..

[73] Nisar S., Shah P.A., Kuchay M.S., Bhat M.A., Rashid A., Ahmed S., Ganie M.A. (2012). Association of polycystic ovary syndrome and Graves’ disease: Is autoimmunity the link between the two diseases. Indian J. Endocrinol. Metab..

[74] Singh R.P., Massachi I., Manickavel S., Singh S., Rao N.P., Hasan S., Mc Curdy D.K., Sharma S., Wong D., Hahn B.H. (2013). The role of miRNA in inflammation and autoimmunity. Autoimmun. Rev..

[75] Xu X., Du C., Zheng Q., Peng L., Sun Y. (2014). Effect of metformin on serum interleukin-6 levels in polycystic ovary syndrome: A systematic review. BMC Women Health.

[76] Ojeda-Ojeda M., Murri M., Insenser M., Escobar-Morreale H.F. (2013). Mediators of low-grade chronic inflammation in polycystic ovary syndrome (PCOS). Curr. Pharm. Des..

[77] Payne G.W. (2006). Effect of inflammation on the aging microcirculation: Impact on skeletal muscle blood flow control. Microcirculation.

[78] Stachenfeld N.S., Taylor H.S. (2004). Effects of estrogen and progesterone administration on extracellular fluid. J. Appl. Physiol..

[79] Machado R.B., Tachotti F., Cavenague G., Maia E. (2006). Effects of two different oral contraceptives on total body water: A randomized study. Contraception.

[80] Franchini M., Caruso C., Nigrelli S., Poggiali C. (1995). Evaluation of body composition during low-dose estrogen oral contraceptives treatment. Acta Eur. Fertil..

[81] Piccoli A., Crosignani P., Nappi C., Ronsini S., Bruni V., Marelli S., Italian E.C.S.G. (2008). Effect of the ethinylestradiol/norelgestromin contraceptive patch on body composition. Results of bioelectrical impedance analysis in a population of Italian women. Nutr. J..

[82] Beberashvili I., Azar A., Sinuani I., Kadoshi H., Shapiro G., Feldman L., Sandbank J., Averbukh Z. (2014). Longitudinal changes in bioimpedance phase angle reflect inverse changes in serum IL-6 levels in maintenance hemodialysis patients. Nutrition.

[83] Dworschak M., Campbell M.J. (2015). About the benefits and limitations of pilot studies. Minerva Anestesiol..

[84] Pin F., Bonetto A., Bonewald L.F., Klein G.L. (2019). Molecular Mechanisms Responsible for the Rescue Effects of Pamidronate on Muscle Atrophy in Pediatric Burn Patients. Front. Endocrinol..

[85] Han Y., Li Y., He B. (2019). GLP-1 receptor agonists versus metformin in PCOS: A systematic review and meta-analysis. Reprod. Biomed. Online.

